# Improved salp swarm algorithm-driven deep CNN for brain tumor analysis

**DOI:** 10.1038/s41598-025-09326-y

**Published:** 2025-07-09

**Authors:** Umang Kumar Agrawal, Nibedan Panda, Ghanshyam G. Tejani, Seyed Jalaleddin Mousavirad

**Affiliations:** 1 School of Computer Engineering, KIIT Deemed to be University, Bhubaneswar, Odisha 751024 India; 2 Department of Research Analytics, Saveetha Institute of Medical and Technical Sciences, Saveetha Dental College and Hospitals, Saveetha University, Chennai, 600077 India; 3 Department of Industrial Engineering and Management, Yuan Ze University, Taoyuan, 320315 Taiwan; 4 Department of Computer and Electrical Engineering, Mid Sweden University, Sundsvall, Sweden

**Keywords:** SSA, Local search, Medical imaging, Prognosis, CNN, Brain MRI, Computational science, Computer science

## Abstract

The efficiency of the swarm-based approach depends on the perfect balance of operators: exploration and exploitation. Due to a lack of balance between these two factors, the Salp Swarm Algorithm (SSA), a recently developed swarm-based metaheuristic approach, suffers from confined minima, stuck and untimely premature convergence. This paper introduces a new, improvised hybrid SSA named Local Search SSA (LS-SSA) to address the pitfalls associated with standard SSA. To prove the competency of the suggested LS-SSA, it is assessed over the twenty-eight functions suite of IEEE-CEC-2017 relating to a diverse set of contemporary methods. Furthermore, a sequence of non-parametric assessments was conducted to establish the statistical significance of the proposed LS-SSA. As a weak exploitation strength for neighbor exploration, SSA may result in less refined parameter tuning of CNN for healthcare-based medical imaging. Hence, LS-SSA is an effective algorithm for hyperparameter tuning of CNNs evaluated on medical imaging datasets, specifically brain MRI. This leads to improved model performance, characterized by higher accuracy, reduced standard deviation, lower minimum RMSE values, and higher average performance. Consequently, optimal candidate solutions with improved and faster convergence toward global optima are achieved.

## Introduction

In the recent past, metaheuristic approaches have emerged to handle real-world high-complexity optimization problems^1^. Optimization is the process of determining the optimum outcome out of a specified pool of candidate solutions optimized through a specific objective function. Nowadays, algorithms with lower time complexity and better convergence rate towards the global optimum are gaining attention as the solution to the optimization problem. The deterministic approach serves as an approachable method for finding the solution, but is deficient in the initial choosing of particles and often gets trapped in the local minima^2^. As a result, metaheuristic approaches evolve as a better alternative, as they follow the stochastic approach. The term metaheuristic is defined as a top-level refined procedure for exploring the candidate solution, which in turn exploits an optimal solution for any complex optimization problems^3^. The distinct features that lead to the higher acceptance of these methods are (i) the way of choosing the initial constraints, (ii) the methodology to undertake the legitimate task, (iii) not problem-specific, and (iv) the relevant black-box approach. The optimization techniques are further classified that is based on (i) pure mathematics and (ii) heuristics. The earlier approach follows some pure mathematics concepts such as linear, quadratic and geometric programming, etc. The latter approach is based on heuristics that iterates a single candidate among a set of pools until the anticipated outcome is obtained. It results in faster execution but gets stuck in the local optima. As an alternative to this single particle-based solution, there exists a population-based optimization approach^4^.

The widespread popularity of metaheuristics approaches based on swarm intelligence (SI) has endorsed incorporating and building the hybrid approach with neural networks^5^. The proper fine-tuning of the hyperparameters results in increased performance and efficacy of the model. These parameters mainly include the dense neuron count, filter size, etc. The recent advancement in the context of medical imaging has led to the employment of CNN, making it more robust for the task of automated analysis and hierarchical extraction of the spatial features of the multimodal imaging data (e.g., CT and MRI scans)^6^. Although CNN is a dominant tool, but lacks adequate parameter selection, is sensitive to artefacts, is computationally intensive and overfits the training data. The downsides of CNN can be mitigated by optimizing the parameters with an SI-based metaheuristic approach such as SSA^7^. The optimistic problems are optimized through this nature-inspired SSA algorithm by the salps in search of food particles inside the deep ocean. The incorporation of SSA furnishes an efficient way of tuning the parameters of CNN that mimic the foregoing behavior of these salps. Hence, SSA serves as a better alternative to the traditional approaches such as grid search or random search but yet suffers from the untimely convergence and confined minima stagnation problem.

The No Free Lunch Theorem (NFL), stated by Wolpert and Macready, suggests that no single underlying algorithm can address all sorts of problems in a similar competent way^8^. Due to the widespread field of research, new stochastic-based approaches are added to the metaheuristic library. As each is characterized by its pros and cons, the Hybrid Algorithm (HA) aims to strengthen the robustness of this approach by integrating multiple approaches such that the weakness of one is compensated for by the other^9^. A singular population-based metaheuristic approach uses the trial-and-error method to compete for the optimum solution inspired by natural phenomena of the birds, but often strays from the confined optima trap and premature convergence. To overcome this issue, mathematical operators (MAO) serve as a crucial formulation method for optimization^10^. MAO provides the structure to guide the solution for the optimistic problem, and hence, incorporating it with SI makes it HA. This HA explores the search region more swiftly with enhanced competence. A nature-inspired SI-based metaheuristic approach, namely SSA, has been preferred in this study due to the foraging behavior of salps in deep water for the food search. In this study, we have considered local search techniques to explore the search region of standard SSA with improved exploitation. As an outcome, the LS boosts the behavioral features of SSA with regard to intensification and diversification, enhances the convergence rate and shores up the suggested model against the local minima stagnation issue. The motive of this study is to observe the behavior changes in terms of exploration, exploitation, complexity analysis, and convergence competence through our experimentation. The IEEE-CEC-2017 problem suite is used to assess the potency of the proposed LS-SSA approach over twenty-eight constrained benchmark suites^11^. Along with this, the outcomes computed from CEC-2017 are substantiated over five contemporary metaheuristics approaches. Simultaneously, the uniqueness of LS-SSA is acclaimed by performing a series of non-parametric assessments^12–14^. Finally, to prove the commendable performance of the suggested LS-SSA, realistic applications such as optimizing the hyperparameters of CNN for enhanced medical image analysis tasks^15^.

The later segments are incorporated as follows: The applications of standard SSA are outlined in Sect. 2. Section 3 provides a systematic study of the original SSA and the LS approach. Section 4 explores the proposed hybrid approach, LS-SSA. The verification of the LS-SSA model considering the present-day method is elaborated in Sect. 5. The competence of the suggested LS-SSA with other modern-day approaches, along with a series of non-parametric significance is elaborated in the same section. Section 6 expands the application of the suggested LS-SSA in hypertuning the parameters of CNN on the benchmark medical imaging dataset. The closing part embraces the results and discussion with the future research plan, as exemplified in Sect. 7.

## Literature review/ research gaps/ background details

This section presents the idea about the SSA and its variants available in the literature, along with their applications in diverse fields of science and engineering. In 2024, Guo et al. introduced a new hybrid MIS-based SSA named SDSSA for diagnosing breast cancer pathology images^16^. The author also assessed their model with recent methodology and validated it using 30 benchmarks function. Jebastine developed the CEXGB-ESSO model for the detection and classification of brain tumors^17^. The authors also equated their developed approach with other contemporary methods. Mahapatra et al. developed a quantized orthogonal hybrid SSA (QOX-SSA) to optimize feature selection and training RBFNN^18^. The author also compared recent algorithms and assessed them with the 30 benchmark functions. In 2023, Abualigah et al. proposed an improved reptile search integrating SSA (RSA-SSA) for medical image segmentation^19^. The authors conducted a non-parametric assessment to evaluate their model. Al-Betar et al. introduced ESSA for training the feed-forward backpropagation neural network (FFNN) for forecasting the cost of software development^20^. The author had also equated with recent algorithms and also assessed the same with the 19 benchmark functions. Alsubai et al. developed the HDLISSA-MGDC model for the detection and classification of Grape disease^21^. The authors also equated their developed method with other contemporary methods. Ehsaeyan introduced EMSSA for medical image segmentation^22^. The authors also equated their developed algorithm with other contemporary methods. Mahapatra et al. developed a quantized SSA (QSSA) to optimize feature selection^23^. The author also compared recent algorithms and also assessed them with the same 30 benchmark functions.

In 2022, Alyami et al. proposed a SSA-based transfer learning model to diagnose brain tumours^24^. The authors also compared their findings with the recent algorithms. Kassaymeh et al. developed an SSA-BPNN to detect software faults^25^. The authors also equated their developed model with other contemporary methods. Syed et al. developed WSSA for sensor deployment^26^. The author also compared recent algorithms and assessed them with the 18 benchmark functions. Wang et al. introduced OOSSA for Mobile robot path planning^27^. The author also assessed their model with recent methodology and validated the same utilizing the 19 benchmarks function. In 2021, Panda and Majhi developed a hybrid model integrating oppositional and mutation operators with SSA (OBL-MO-SSA) for training the FLANN for optimal solution^28^. The author also conducted a non-parametric evaluation to assess the superiority of the model and validated their model by the 28 benchmark constraints. The same author proposed HONN-based SSA to optimize the parameters of PSNN for classification tasks^29^. The authors also compared their model with other present-day approaches. In 2020, the same author proposed a space transformation search-based SSA (STS-SSA) for training the multi-layer perceptron^30^. The author also conducted a non-parametric evaluation to assess the superiority of the model and validated their model by the 28 benchmark constraints. The literature discussed above related to machine learning (ML), deep learning (DL) and hybrid optimization algorithms (OA), along with their applied areas, is presented in Table 1.

**Table 1:** Applications related to ML, DL and hybrid OA-driven methods.

Reference	Algorithm used	Application area	Year
(A) ML-based approach			
Al-Betar et al.^20^	ESSA	Forecasting software development costs	2023
Alsubai et al.^21^	HDLISSA-MGDC	Grape disease classification	2023
Kassaymeh et al.^25^	SSA-BPNN	Software fault prediction problem	2022
Syed et al.^26^	WSSA	Sensor deployment	2022
Wang et al.^27^	OOSSA	Mobile robot path planning	2022
(B) DL-based approach			
Guo et al.^16^	SDSSA	Medical Imaging-Breast cancer	2024
Jebastine^17^	CEXGB-ESSO	Medical Imaging-Brain MRI	2024
Abualigah et al.^19^	RSA-SSA	Medical Image Segmentation	2023
Ehsaeyan^22^	EMSSA	Medical Image Segmentation	2023
Alyami et al.^24^	SVM, SSA	Medical Imaging-Brain MRI	2022
(C) OA-driven technique			
Mahapatra et al.^18^	QOX-SSA	Feature Selection Training RBFNN	2025
Mahapatra et al.^23^	QSSA	Feature Selection	2023
Panda and Majhi^28^	OBL-MO-SSA	Training FLANN	2021
Panda and Majhi^29^	SSA-HONN	Training PSNN	2021
Panda and Majhi^30^	STS-SSA	Training MLP	2020

## Basic preliminaries

The ongoing subsection represents the notion behind SSA, the collective intelligence of animals like salps, and its utilization in research for resolving optimization problems.

### Standard SSA

SSA is one of the most newly developed swarm-based metaheuristic methods added to the nature-inspired evolutionary algorithm literature. The idea behind the SSA was established by Mirjalili et al. in the year of 2017^31^. The prime notion of the SSA was coined from the propulsion and scavenging pattern of the salps inside the deep ocean. The physical body structure of the salps is barrel-shaped and transparent, and fits to Salpidae family. Individual salps are linked with each other and form a chain-like architecture, which is termed a salp chain or salp swarm for their livelihood. The entire populace in the salp chain can be characterized into two classes, as leader and follower. The salp, which is present at the front of the chain, is considered the leader salp and leads the swarm, and the rest are follower salps. The leader salp explores the region by updating its position, conferring towards the foodstuff. Simultaneously, the follower salp in the chain updates their positions sequentially by one another. The position update of the leader salp is represented mathematically in Eq. 1.

1 $$\:{A}_{p}^{1}=\left\{\begin{array}{c}{F}_{p}+\:{P}_{1}\left(\left({HB}_{p}-{LB}_{p}\right){P}_{2}+{LB}_{p}\right){P}_{3}\ge\:0.5\\\:{F}_{p}-\:{P}_{1}\left(\left({HB}_{p}-{LB}_{p}\right){P}_{2}+{LB}_{p}\right){P}_{3}<0.5\end{array}\right.$$

$\:{A}_{p}^{1}$ represents the position of the leader in $\:p$^th^ dimension. $\:{F}_{p}$ represents the position of foodstuff in $\:p$^th^ dimension. $\:{HB}_{p}$ represents a higher bound in $\:p$^th^ dimension. $\:{LB}_{p}$ represents a lower bound in $\:p$^th^ dimension. $\:{P}_{1}$ indicates the most crucial parameter and is responsible for balancing the exploration and exploitation, as defined in Eq. 2.

2 $$\:{P}_{1}={2e}^{-{\left(\frac{y}{Y}\right)}^{2}}$$

$\:y$ signifies the current iteration, and $\:Y$ signifies the total number of iterations. $\:{P}_{2}\:\text{a}\text{n}\text{d}\:{P}_{3}$ signify two random variables in the range between 0 and 1. The position update of the follower salp is represented mathematically in Eq. 3.

3 $$\:{a}_{p}^{k}=\frac{1}{2}\left({a}_{p}^{k}+{a}_{p}^{k-1}\right)$$

$\:{a}_{p}^{k}$ represents the location of the $\:k$^th^ salp in the $\:p$^th^ dimension. $\:{a}_{p}^{k-1}$ represents the location of the $\:\left(k-1\right)$^th^ salp in the $\:p$^th^ dimension. According to optimization, the entire algorithm will be considered as the colony of salps, where the scavengers are treated as search agents, and the ocean is the search space. The exploration and exploitation process will be carried out by the leader salp throughout the search space by updating its position according to the foodstuff and simultaneously saving the result obtained from individual iterations. The final optimum solution is considered the global optimum outcome after successful computation of all iterations. The SSA algorithm shows supremacy over other recent metaheuristic algorithms such as (i) only one controlling factor, $\:{P}_{1}$ which is lessened linearly (ii) it saves the optimum outcome observed in individual iteration with the help of a food source variable for further use if the entire population collapses also (iii) leader salp explores the entire space by updating its position according to the foodstuff and follower salps change their locations by each other and consciously move near leader, which results in avoiding the SSA to stagnate in local optima. The pseudocode representation of SSA is presented in Table 2.

**Table 2:**
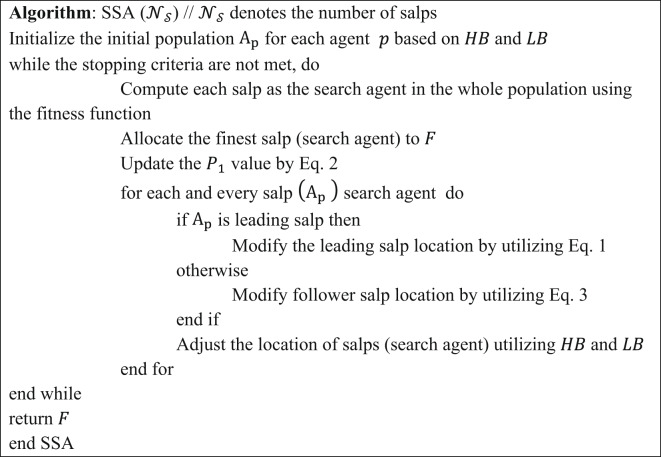
Pseudocode representation of SSA^31^.

### The local search technique

The local search-based technique is generally a heuristic approach that attempts to optimize the local optima stagnation issue. It enhances the candidate particles by exploring the solution within the specified range of the neighbor domain. It has localized search particles that randomly perturb in the search space of the given radius $\:{\mathcal{R}}_{\text{p}}$ and updates the particles $\:{\mathcal{b}}_{\text{p}}$ with the best score for the objective function. This refinement procedure helps the optimization algorithm with global exploration, faster convergence rate and reducing the risk for issue of confined optima.

4 $$\:{\mathcal{R}}_{\mathcal{i}}=0.05\:\text{*}\left|HB-\:LB\:\right|$$

5 $$\:{\mathcal{b}}_{\text{p}}\:={\text{A}}_{\text{p}}+{\upgamma\:}$$

6 $$\:{\upgamma\:}\: \epsilon\: ({-\mathcal{R}}_{\mathcal{i}}+{\mathcal{R}}_{\mathcal{i}})$$

## The proposed approach: improved SSA integrating local search (LS-SSA)

**Fig. 1:**
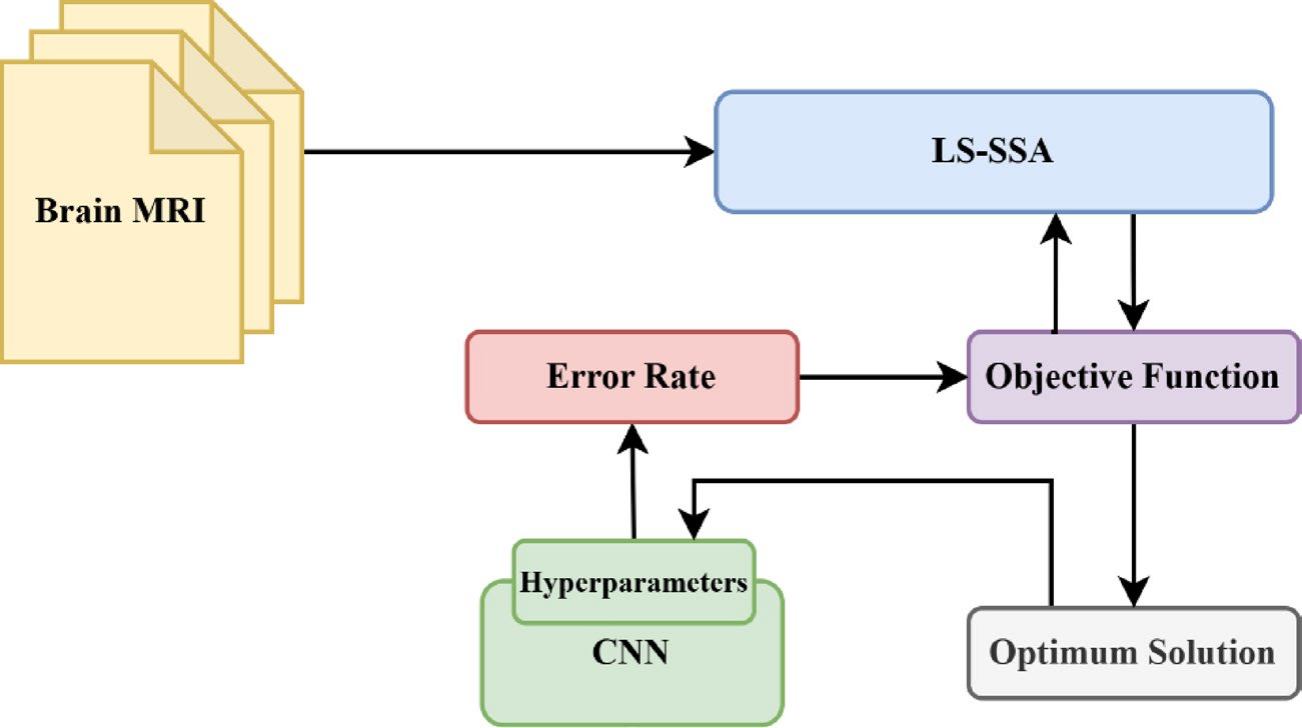
Flow graph of suggested LS-SSA optimizing CNN.

The prime concept beyond the development of the proposed improved SSA, unified with a local search technique termed LS-SSA, is described in this section. The foremost emphasis is to leverage the explorative potential of the search agents congenital in the standard SSA. Swarm-based approaches are governed by two parameters, namely exploration and exploitation. The pitfalls in the basic SSA include the unbalance between the above parameters, poor convergence, confined optima, stuck, premature convergence and lack of adaptive properties, which are regularly fine-tuned through these localized search agents. A local search is a fruitful approach that works iteratively to find the optimal solution by exploring the neighbor bounds. It begins exploration with the initial positions and continues till it gets the optimal solution for the salp or the maximum range of iterations. The main focus of LS is to explore the local optimum within the stipulated time frame, as it can proficiently discover the confined candidate within the salp’s search space. In this experimentation, the search space is exploited within the confined radius of the initial salp chosen. To achieve the same, 5% of the bounds of the salp position is chosen as the search radius. The leader salp is obtained by concurrently perturbing according to the food source within the search space. The individual salps exploit the region in subsequent iterations. Hence, we may expect the suggested approach to attain the global optimum, which is more specific and superior. The suggested LS-SSA can escape stagnation in confined minima by regularly refining the candidate solution, making the metaheuristic approaches more robust and making a perfect match between the exploration and exploitation. Thus, the unification of LS with SSA can perform local refinements and result in faster and smoother convergence towards the global optimum. The efficacy of LS-SSA is tuned by one determinant factor, which is depicted as $\:{\upgamma\:}.$ The factor assists the suggested approach in escaping the confined minima stuck by linearly exploiting its neighbor bounds, hence defining a perilous role in the intensification and diversification of the exploration domain. The pseudocode representation of LS-SSA is reported in Table 3. The flow graph of the suggested LS-SSA optimizing CNN is depicted in Fig. 1.

**Table 3:**
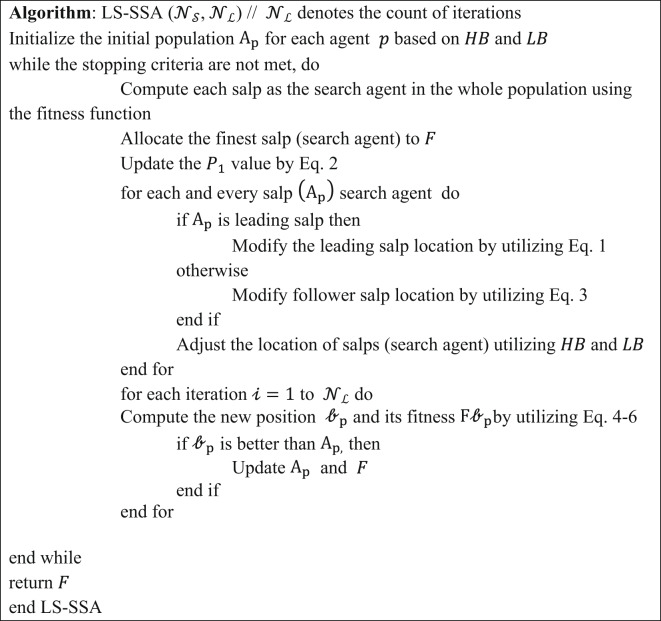
Pseudocode representation of LS-SSA.

## Experimentation setting and outcome analysis

A robust facility is needed for the exploration of the refinement capacity of the suggested algorithm, considering the extensive medical image dataset, stimulation and computational jobs. A high-performance system having the configuration of 12th Gen Intel(R) Core(TM) i7-12650HX @ 4.8 GHz mounted with 16 GB of RAM and 4 GB of GTX 1650, has been utilized to run the simulation.

### Outcome analysis

The proposed LS-SSA approach has been assessed using the benchmark suite included in the IEEE-CEC-2017 to validate the effectiveness of the LS boosted SSA. In this segment, SSA and LS-SSA have been assessed, and the results are obtained using 100 iterations over magnitudes 10 and 30, as reported in Tables 4 and 5. The CEC-2017 entails twenty-eight functions specifically unimodal (f1-f3), multimodal (f4-f10), hybrid and composite (f11-f28). Unimodal explores the perturbing nature of the stochastic methods, multimodal inspects the model’s power to escape the local optima stuck, whereas the hybrid functions inspect the suggested LS-SSA over the confined optima problem. The area of the exploration lies within the extent of [-10,10] and [-30,30], although retaining the particle size fixed to 30. The performative access unified in the CEC-2017 is represented as standard deviation (SD), mean, minimal, maximal and median. The proficiency for attaining the global optimum is predominated by the improved LS-SSA over the original SSA. The superior results are indicated in bold. The parameters and their values are presented in Table 6.

**Table 4:** Parameters acquired by SSA and LS-SSA over objective functions pertaining to the CEC-2017 suite with magnitude 10.

Function	Approach	Mean	Median	SD	Minimal	Maximal
f1	SSA	2.452E + 03	2.280E + 03	1.133E + 03	1.005E + 03	5.423E + 03
	LS-SSA	**2.689E + 02**	2.280E + 02	1.157E + 02	1.452E + 02	5.100E + 02
f2	SSA	4.160E + 08	1.055E + 08	4.727E + 08	4.610E + 04	1.186E + 09
	LS-SSA	**3.444E + 08**	6.491E + 05	1.005E + 09	1.418E + 04	3.358E + 09
f3	SSA	4.003E + 03	2.588E + 03	2.592E + 03	1.697E + 03	9.564E + 03
	LS-SSA	**1.344E + 03**	1.221E + 03	7.235E + 02	3.419E + 02	2.873E + 03
f4	SSA	3.064E + 01	3.001E + 01	6.198E + 00	2.084E + 01	4.193E + 01
	LS-SSA	**1.538E + 01**	1.582E + 01	4.326E + 00	6.418E + 00	2.084E + 01
f5	SSA	2.222E + 08	1.234E + 08	2.413E + 08	7.643E + 06	7.532E + 08
	LS-SSA	**2.102E + 06**	1.772E + 06	2.115E + 06	1.601E + 05	7.983E + 06
f6	SSA	4.591E + 03	4.380E + 03	2.364E + 03	1.018E + 03	7.741E + 03
	LS-SSA	**2.940E + 02**	1.986E + 02	1.924E + 02	9.922E + 01	7.609E + 02
f7	SSA	8.783E + 06	6.596E + 06	7.662E + 06	1.719E + 06	2.561E + 07
	LS-SSA	**1.403E + 05**	9.280E + 04	1.193E + 05	1.106E + 04	3.914E + 05
f8	SSA	-2.740E + 02	-2.817E + 02	5.427E + 01	-3.912E + 02	-1.862E + 02
	LS-SSA	**-4.470E + 02**	-4.362E + 02	6.757E + 01	-5.532E + 02	-3.540E + 02
f9	SSA	2.883E + 03	2.952E + 03	7.466E + 02	1.827E + 03	3.942E + 03
	LS-SSA	**4.884E + 02**	3.646E + 02	3.102E + 02	2.417E + 02	1.338E + 03
f10	SSA	2.060E + 01	2.061E + 01	2.409E-01	2.019E + 01	2.095E + 01
	LS-SSA	**1.973E + 01**	2.050E + 01	2.255E + 00	1.299E + 01	2.077E + 01
f11	SSA	1.758E + 00	1.705E + 00	3.297E-01	1.163E + 00	2.320E + 00
	LS-SSA	**1.127E + 00**	1.125E + 00	1.185E-01	9.500E-01	1.425E + 00
f12	SSA	5.123E + 07	3.016E + 07	6.082E + 07	3.526E + 06	2.159E + 08
	LS-SSA	**3.537E + 04**	4.046E + 03	8.046E + 04	1.335E + 01	2.717E + 05
f13	SSA	1.045E + 08	5.875E + 07	1.715E + 08	1.128E + 06	6.076E + 08
	LS-SSA	**1.178E + 05**	3.175E + 04	1.877E + 05	1.430E + 04	6.615E + 05
f14	SSA	**1.593E-02**	1.095E-02	1.186E-02	6.886E-04	3.565E-02
	LS-SSA	7.152E-02	5.380E-02	4.917E-02	1.310E-02	1.617E-01
f15	SSA	9.753E-01	1.197E-01	1.282E + 00	7.922E-03	4.099E + 00
	LS-SSA	**1.936E-01**	1.104E-02	4.787E-01	2.048E-03	1.617E + 00
f16	SSA	9.101E + 01	8.022E + 01	8.019E + 01	3.211E + 00	2.525E + 02
	LS-SSA	**6.227E + 00**	4.579E + 00	4.546E + 00	2.595E + 00	1.818E + 01
f17	SSA	5.608E + 03	4.883E + 03	2.686E + 03	1.638E + 03	1.017E + 04
	LS-SSA	**3.215E + 02**	3.221E + 02	1.333E + 02	1.442E + 02	5.449E + 02
f18	SSA	6.093E + 01	5.850E + 01	1.366E + 01	4.441E + 01	8.507E + 01
	LS-SSA	**5.023E + 01**	4.181E + 01	2.243E + 01	2.526E + 01	8.820E + 01
f19	SSA	6.509E + 01	6.646E + 01	2.241E + 01	3.007E + 01	9.450E + 01
	LS-SSA	**4.830E + 01**	4.865E + 01	1.672E + 01	1.900E + 01	8.276E + 01
f20	SSA	9.626E + 04	8.648E + 04	6.478E + 04	3.660E + 04	2.748E + 05
	LS-SSA	**2.556E + 03**	1.809E + 03	1.933E + 03	9.634E + 02	7.191E + 03
f21	SSA	2.139E + 08	2.158E + 08	1.220E + 08	1.595E + 07	4.577E + 08
	LS-SSA	**1.235E + 06**	7.041E + 05	1.211E + 06	1.948E + 05	3.587E + 06
f22	SSA	7.226E + 16	1.969E + 16	8.191E + 16	4.708E + 14	2.389E + 17
	LS-SSA	**2.487E + 13**	1.468E + 12	4.758E + 13	6.991E + 10	1.418E + 14
f23	SSA	-4.370E + 02	-4.052E + 02	1.519E + 02	-7.321E + 02	-2.041E + 02
	LS-SSA	**-4.709E + 02**	-4.674E + 02	1.075E + 02	-6.306E + 02	-2.224E + 02
f24	SSA	1.777E + 03	1.740E + 03	4.350E + 02	1.170E + 03	2.555E + 03
	LS-SSA	**8.837E + 02**	8.767E + 02	5.627E + 02	1.105E + 02	1.889E + 03
f25	SSA	1.860E + 02	1.916E + 02	5.241E + 01	1.136E + 02	2.641E + 02
	LS-SSA	**1.127E + 02**	9.394E + 01	6.407E + 01	6.161E + 01	2.874E + 02
f26	SSA	-3.485E + 03	-2.251E + 03	3.262E + 03	-1.223E + 04	-4.893E + 02
	LS-SSA	**-3.754E + 03**	-2.702E + 03	3.155E + 03	-1.198E + 04	-1.111E + 03
f27	SSA	1.176E + 04	1.045E + 04	5.244E + 03	4.991E + 03	2.175E + 04
	LS-SSA	**1.692E + 03**	1.132E + 03	1.338E + 03	4.601E + 02	4.221E + 03
f28	SSA	-3.459E + 00	-3.410E + 00	4.511E-01	-4.272E + 00	-2.779E + 00
	LS-SSA	**-4.638E + 00**	-4.456E + 00	6.439E-01	-5.756E + 00	-3.870E + 00

**Table 5:** Parameters acquired by SSA and LS-SSA over objective functions pertaining to the CEC-2017 suite with magnitude 30.

Function	Approach	Mean	Median	SD	Minimal	Maximal
f1	SSA	1.182E + 04	1.174E + 04	3.220E + 03	4.699E + 03	1.597E + 04
	LS-SSA	**4.470E + 03**	4.330E + 03	9.855E + 02	3.246E + 03	5.829E + 03
f2	SSA	1.005E + 33	1.941E + 29	3.010E + 33	1.546E + 23	1.003E + 34
	LS-SSA	**7.615E + 27**	9.452E + 25	1.491E + 28	5.408E + 23	4.390E + 28
f3	SSA	2.920E + 04	2.556E + 04	1.491E + 04	1.198E + 04	6.304E + 04
	LS-SSA	**2.035E + 04**	2.058E + 04	6.618E + 03	9.409E + 03	2.998E + 04
f4	SSA	4.279E + 01	4.206E + 01	4.168E + 00	3.569E + 01	4.981E + 01
	LS-SSA	**3.814E + 01**	3.737E + 01	7.784E + 00	2.822E + 01	5.103E + 01
f5	SSA	1.484E + 09	1.079E + 09	9.487E + 08	4.428E + 08	3.048E + 09
	LS-SSA	**2.667E + 08**	2.797E + 08	1.147E + 08	1.021E + 08	4.254E + 08
f6	SSA	1.117E + 04	1.067E + 04	2.796E + 03	7.205E + 03	1.838E + 04
	LS-SSA	**5.490E + 03**	4.822E + 03	1.780E + 03	3.695E + 03	9.483E + 03
f7	SSA	2.303E + 08	1.285E + 08	2.606E + 08	4.446E + 07	9.637E + 08
	LS-SSA	**3.467E + 07**	3.605E + 07	1.684E + 07	1.020E + 07	7.362E + 07
f8	SSA	-4.157E + 02	-4.251E + 02	6.941E + 01	-5.322E + 02	-3.096E + 02
	LS-SSA	**-9.706E + 02**	-9.823E + 02	8.948E + 01	-1.097E + 03	-8.049E + 02
f9	SSA	1.322E + 04	1.227E + 04	4.044E + 03	7.601E + 03	2.181E + 04
	LS-SSA	**4.986E + 03**	4.790E + 03	1.417E + 03	2.604E + 03	7.687E + 03
f10	SSA	2.083E + 01	2.076E + 01	2.025E-01	2.052E + 01	2.116E + 01
	LS-SSA	**2.077E + 01**	2.087E + 01	4.389E-01	1.955E + 01	2.119E + 01
f11	SSA	3.910E + 00	3.907E + 00	5.088E-01	3.005E + 00	4.703E + 00
	LS-SSA	**2.595E + 00**	2.765E + 00	6.013E-01	1.745E + 00	3.621E + 00
f12	SSA	2.658E + 08	2.317E + 08	1.292E + 08	9.003E + 07	5.276E + 08
	LS-SSA	**1.015E + 08**	8.368E + 07	8.176E + 07	1.249E + 07	2.654E + 08
f13	SSA	7.970E + 08	7.792E + 08	4.905E + 08	2.273E + 08	2.020E + 09
	LS-SSA	**1.763E + 08**	8.045E + 07	1.552E + 08	3.362E + 07	4.788E + 08
f14	SSA	**6.294E-03**	5.295E-03	3.440E-03	2.492E-03	1.380E-02
	LS-SSA	6.070E-02	4.643E-02	3.982E-02	5.466E-03	1.267E-01
f15	SSA	6.831E + 00	3.885E-01	1.150E + 01	1.440E-02	3.780E + 01
	LS-SSA	**2.067E-02**	1.010E-02	3.046E-02	1.940E-03	1.093E-01
f16	SSA	6.256E + 02	2.892E + 02	8.239E + 02	8.339E + 00	2.803E + 03
	LS-SSA	**1.229E + 01**	1.081E + 01	7.283E + 00	2.765E + 00	2.772E + 01
f17	SSA	2.247E + 04	2.269E + 04	4.588E + 03	1.394E + 04	3.068E + 04
	LS-SSA	**9.745E + 03**	9.933E + 03	2.154E + 03	6.099E + 03	1.313E + 04
f18	SSA	2.675E + 02	2.693E + 02	4.778E + 01	1.628E + 02	3.468E + 02
	LS-SSA	**2.178E + 02**	2.135E + 02	4.023E + 01	1.328E + 02	2.744E + 02
f19	SSA	2.439E + 02	2.477E + 02	2.770E + 01	2.024E + 02	2.933E + 02
	LS-SSA	**2.049E + 02**	1.968E + 02	2.760E + 01	1.617E + 02	2.561E + 02
f20	SSA	4.432E + 05	4.202E + 05	1.163E + 05	2.449E + 05	7.019E + 05
	LS-SSA	**1.151E + 05**	1.053E + 05	5.610E + 04	4.465E + 04	2.546E + 05
f21	SSA	1.198E + 09	1.093E + 09	5.136E + 08	3.007E + 08	2.037E + 09
	LS-SSA	**3.927E + 08**	3.890E + 08	1.827E + 08	1.257E + 08	6.367E + 08
f22	SSA	8.605E + 17	6.609E + 17	7.807E + 17	1.002E + 17	2.835E + 18
	LS-SSA	**4.904E + 16**	4.233E + 16	3.488E + 16	9.161E + 15	1.338E + 17
f23	SSA	-1.162E + 03	-1.193E + 03	2.320E + 02	-1.523E + 03	-8.615E + 02
	LS-SSA	**-1.303E + 03**	-1.211E + 03	3.385E + 02	-1.904E + 03	-7.262E + 02
f24	SSA	1.009E + 04	1.009E + 04	2.393E + 03	6.561E + 03	1.374E + 04
	LS-SSA	**8.587E + 03**	8.323E + 03	1.839E + 03	5.707E + 03	1.269E + 04
f25	SSA	5.990E + 02	6.020E + 02	6.084E + 01	4.878E + 02	6.939E + 02
	LS-SSA	**5.079E + 02**	5.048E + 02	7.876E + 01	3.729E + 02	6.175E + 02
f26	SSA	-2.899E + 09	-6.704E + 07	5.672E + 09	-1.887E + 10	-3.878E + 06
	LS-SSA	**-2.813E + 10**	-2.349E + 08	8.222E + 10	-2.748E + 11	-7.735E + 06
f27	SSA	1.849E + 05	1.842E + 05	3.610E + 04	1.387E + 05	2.651E + 05
	LS-SSA	**7.944E + 04**	8.074E + 04	9.564E + 03	6.694E + 04	9.579E + 04
f28	SSA	**-7.573E + 00**	-6.851E + 00	1.642E + 00	-1.041E + 01	-5.407E + 00
	LS-SSA	-7.305E + 00	-7.233E + 00	6.086E-01	-8.305E + 00	-6.160E + 00

**Table 6:** Parameters and their values.

Parameters	Values
population size	30
iteration count	100
learning rates	0.05
dimension	10 and 30
CNN’s hyperparameter range	dense neurons count - [16,64] filter count - [64,256]

### Consideration with contemporary metaheuristics approach

Present-day metaheuristic algorithms deliver the path to explore the global optimum for a problem suite timelessly. Diversified approaches are recognized for their varied characteristic to solve the optimization problem. Controlling parameters regulate the exploration facility of these methods. The comparison of these approaches with that of LS-SSA over the expanded number of controlling parameters, along with convergence towards the optima, has gained some impression. Henceforth, we have outlined a fair assessment of the underlying six approaches along with LS-SSA over the problem suite of CEC-2017, having magnitudes 10 and 30. The maximum count of iterations taken into account is (10^2^ * specified dimensions) for the individual function suite. The present-day methods, together with the manifest LS-SSA approach, additionally ALO^32^, MFO^33^, SCA^34^, WOA^35^, and SSA have been considered for analysis of the contemporary approaches. From the results reported in Tables 7 and 8, LS-SSA confirms its predominance over the present-day approaches.

**Table 7:** Mean value assessment of present-day approaches over the IEEE-CEC-2017 suite with dimension 10.

	ALO	MFO	SCA	WOA	SSA	LS-SSA
f1	1.762E + 04	1.527E + 04	1.450E + 04	3.485E + 03	2.452E + 03	**2.689E + 02**
f2	1.714E + 13	7.095E + 11	2.913E + 11	9.486E + 09	4.160E + 08	**3.444E + 08**
f3	2.010E + 04	1.882E + 04	1.734E + 04	1.940E + 04	4.003E + 03	**1.344E + 03**
f4	7.376E + 01	6.775E + 01	6.871E + 01	4.746E + 01	3.064E + 01	**1.538E + 01**
f5	4.508E + 09	3.998E + 09	4.608E + 09	2.684E + 08	2.222E + 08	**2.102E + 06**
f6	1.570E + 04	1.628E + 04	1.185E + 04	3.187E + 03	4.591E + 03	**2.940E + 02**
f7	1.980E + 08	1.848E + 08	1.555E + 08	3.572E + 07	8.783E + 06	**1.403E + 05**
f8	-2.717E + 02	-2.115E + 02	-4.009E + 02	**-5.607E + 02**	-2.740E + 02	-4.470E + 02
f9	1.461E + 04	1.462E + 04	1.398E + 04	3.033E + 03	2.883E + 03	**4.884E + 02**
f10	2.022E + 01	2.088E + 01	2.005E + 01	**6.377E + 00**	2.060E + 01	1.973E + 01
f11	5.112E + 00	4.881E + 00	4.691E + 00	1.593E + 00	1.758E + 00	**1.127E + 00**
f12	3.278E + 09	2.001E + 09	3.573E + 09	6.234E + 07	5.123E + 07	**3.537E + 04**
f13	4.158E + 09	4.070E + 09	2.887E + 09	2.426E + 08	1.045E + 08	**1.178E + 05**
f14	7.518E-03	1.309E-01	**6.721E-03**	1.000E-02	1.593E-02	7.152E-02
f15	1.573E + 00	3.087E + 01	4.195E-01	6.969E-01	9.753E-01	**1.936E-01**
f16	6.321E + 02	6.980E + 02	3.826E + 02	5.115E + 02	9.101E + 01	**6.227E + 00**
f17	2.091E + 04	1.988E + 04	1.438E + 04	4.271E + 08	5.608E + 03	**3.215E + 02**
f18	1.805E + 02	1.298E + 02	1.322E + 02	7.223E + 01	6.093E + 01	**5.023E + 01**
f19	1.720E + 02	1.475E + 02	1.465E + 02	6.999E + 01	6.509E + 01	**4.830E + 01**
f20	8.942E + 05	9.093E + 05	7.218E + 05	6.012E + 04	9.626E + 04	**2.556E + 03**
f21	5.502E + 09	3.499E + 09	6.836E + 09	5.965E + 08	2.139E + 08	**1.235E + 06**
f22	1.544E + 19	1.779E + 19	1.064E + 19	3.318E + 17	7.226E + 16	**2.487E + 13**
f23	-5.280E + 02	-3.933E + 02	-5.253E + 02	**-8.298E + 02**	-4.370E + 02	-4.709E + 02
f24	1.262E + 04	1.155E + 04	8.200E + 03	2.081E + 03	1.777E + 03	**8.837E + 02**
f25	4.061E + 02	3.453E + 02	3.590E + 02	1.984E + 02	1.860E + 02	**1.127E + 02**
f26	-1.065E + 04	-9.163E + 02	-2.693E + 04	**-3.462E + 04**	-3.485E + 03	-3.754E + 03
f27	7.153E + 04	7.092E + 04	4.677E + 04	1.025E + 04	1.176E + 04	**1.692E + 03**
f28	-3.727E + 00	-4.052E + 00	-3.774E + 00	**-8.727E + 00**	-3.459E + 00	-4.638E + 00

**Table 8:** Mean value assessment of present-day approaches over the IEEE-CEC-2017 suite with dimension 30.

	ALO	MFO	SCA	WOA	SSA	LS-SSA
f1	7.211E + 04	6.932E + 04	7.355E + 04	8.206E + 03	1.182E + 04	**4.470E + 03**
f2	1.860E + 43	7.863E + 41	1.058E + 37	1.572E + 30	1.005E + 33	**7.615E + 27**
f3	1.531E + 05	1.170E + 05	1.290E + 05	1.485E + 05	2.920E + 04	**2.035E + 04**
f4	8.946E + 01	8.609E + 01	9.029E + 01	4.739E + 01	4.279E + 01	**3.814E + 01**
f5	3.361E + 10	3.230E + 10	3.466E + 10	2.866E + 09	1.484E + 09	**2.667E + 08**
f6	6.971E + 04	6.325E + 04	7.327E + 04	1.234E + 04	1.117E + 04	**5.490E + 03**
f7	4.642E + 09	4.155E + 09	4.772E + 09	2.897E + 08	2.303E + 08	**3.467E + 07**
f8	-4.893E + 02	-5.376E + 02	-6.191E + 02	-1.562E + 03	-4.157E + 02	**-9.706E + 02**
f9	7.028E + 04	6.599E + 04	7.574E + 04	1.171E + 04	1.322E + 04	**4.986E + 03**
f10	2.055E + 01	2.126E + 01	2.045E + 01	**1.157E + 01**	2.083E + 01	2.077E + 01
f11	1.776E + 01	1.807E + 01	1.863E + 01	3.822E + 00	3.910E + 00	**2.595E + 00**
f12	1.988E + 10	1.703E + 10	2.255E + 10	1.252E + 09	2.658E + 08	**1.015E + 08**
f13	2.377E + 10	2.441E + 10	2.849E + 10	4.074E + 08	7.970E + 08	**1.763E + 08**
f14	1.012E-02	2.807E-02	8.815E-03	8.951E-03	**6.294E-03**	6.070E-02
f15	1.509E + 00	8.205E + 01	1.636E-01	6.150E-02	6.831E + 00	**2.067E-02**
f16	1.367E + 03	5.382E + 02	4.488E + 02	1.656E + 03	6.256E + 02	**1.229E + 01**
f17	1.161E + 05	9.835E + 04	1.124E + 05	9.671E + 08	2.247E + 04	**9.745E + 03**
f18	7.404E + 02	6.122E + 02	5.639E + 02	**1.853E + 02**	2.675E + 02	2.178E + 02
f19	7.465E + 02	5.992E + 02	6.268E + 02	2.317E + 02	2.439E + 02	**2.049E + 02**
f20	4.750E + 06	4.685E + 06	5.307E + 06	6.525E + 05	4.432E + 05	**1.151E + 05**
f21	3.115E + 10	2.851E + 10	3.767E + 10	4.457E + 09	1.198E + 09	**3.927E + 08**
f22	1.302E + 20	1.123E + 20	1.564E + 20	5.205E + 18	8.605E + 17	**4.904E + 16**
f23	-1.273E + 03	-8.157E + 02	-1.312E + 03	**-2.536E + 03**	-1.162E + 03	-1.303E + 03
f24	5.850E + 04	5.967E + 04	6.313E + 04	**4.988E + 03**	1.009E + 04	8.587E + 03
f25	1.497E + 03	1.482E + 03	1.450E + 03	**4.317E + 02**	5.990E + 02	5.079E + 02
f26	-6.903E + 10	-2.709E + 06	-4.881E + 11	-1.691E + 13	-2.899E + 09	**-2.813E + 10**
f27	9.105E + 05	8.920E + 05	9.380E + 05	1.648E + 05	1.849E + 05	**7.944E + 04**
f28	-7.158E + 00	-6.019E + 00	-6.611E + 00	-2.732E + 01	**-7.573E + 00**	-7.305E + 00

### Convergence analysis

**Fig. 2:**
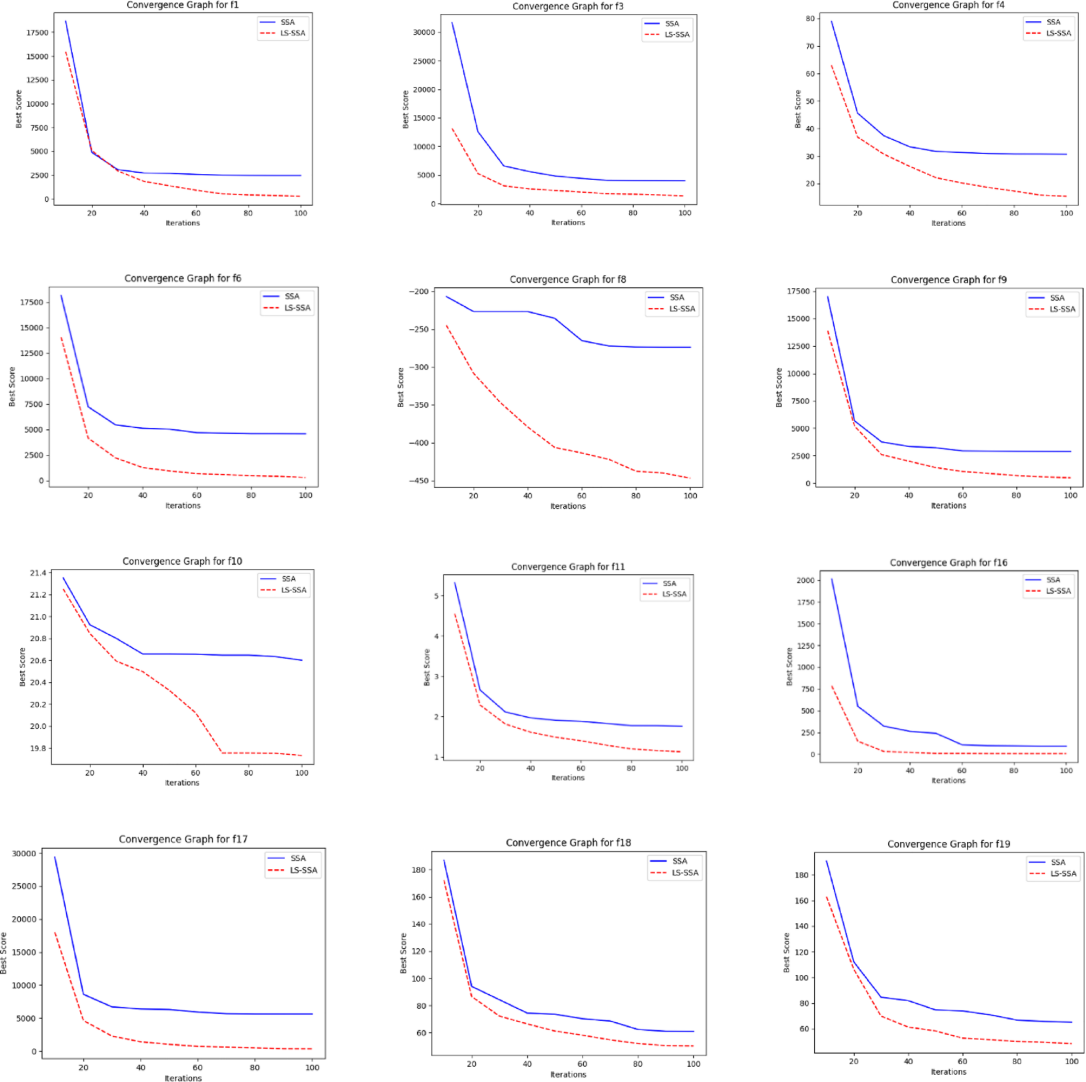

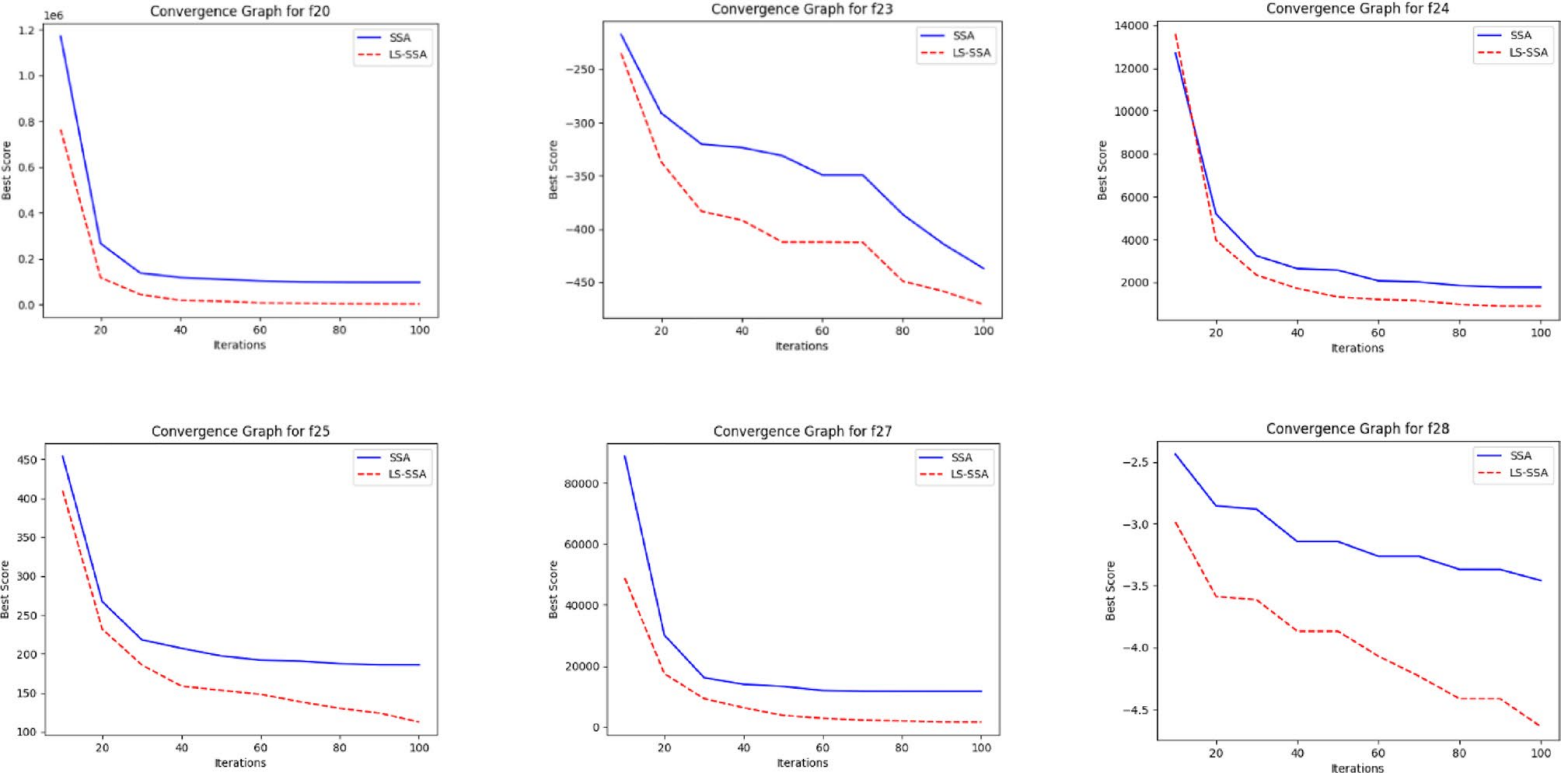
Convergence graphs obtained from the suite of IEEE-CEC-2017 having dimension 10.

**Fig. 3:**
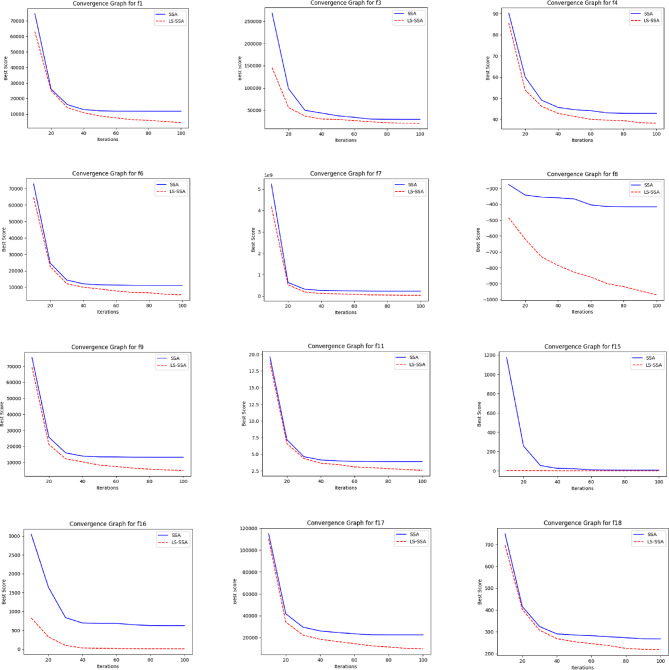

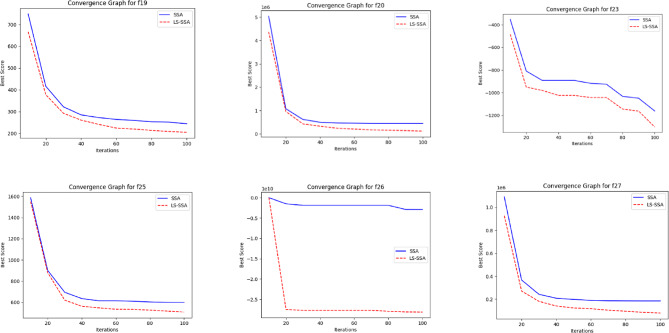
Convergence graphs obtained from the suite of IEEE-CEC-2017 having dimension 30.

**Fig. 4:**
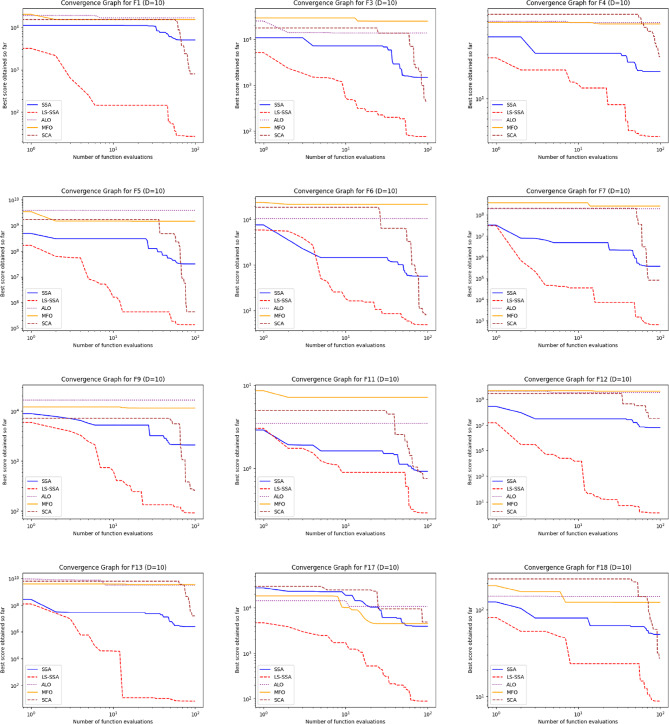

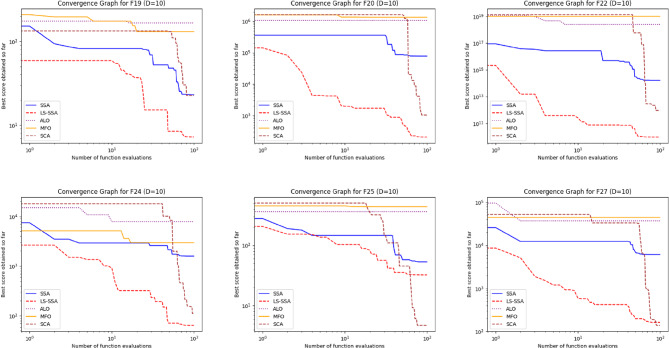
Function evaluation graphs obtained from the suite of IEEE-CEC-2017 having dimension 10 over the considered OA.

**Fig. 5:**
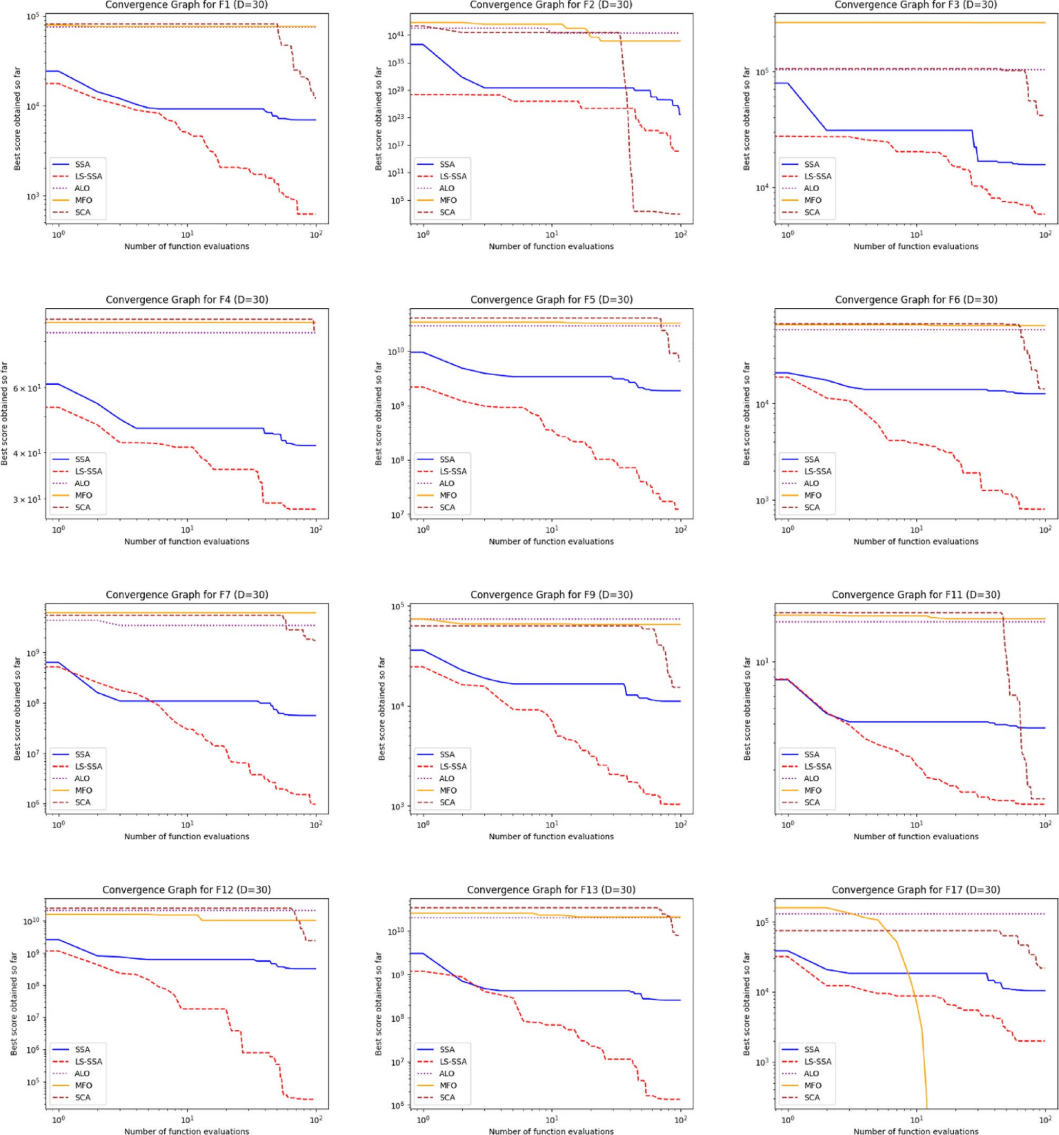

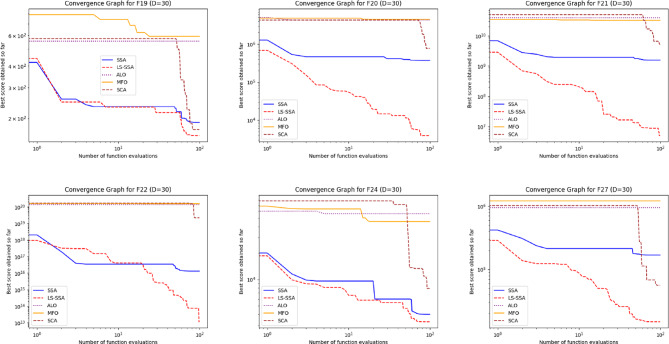
Function evaluation graphs obtained from the suite of IEEE-CEC-2017 having dimension 30 over the considered OA.

The development of the swarm-based approaches involves the phenomenon of convergence. For every subsequent phase of an iterative computational technique, there will be a solution to which it moves near the anticipated result. The convergence happenings of the suggested LS-SSA and standard SSA method over the standardized suite of CEC-2017, having the magnitudes 10 and 30, are presented in Figs. 2 and 3. The horizontal axis outlines reiterations count, and the vertical axis depicts the benchmark constraints ethics of the said approaches. From the stated outcome, it can be perceived that LS-SSA is more improved than SSA due to its untimely convergences towards the global optima without the confined optima entrapment. The function evaluation graphs obtained from the suite of IEEE-CEC-2017, having dimensions 10 and 30 over the considered OA are presented in Figs. 4 and 5.

### Non-parametric assessment

This sub-section assesses the statistical competency of the proposed LS-SSA approach based on outcomes obtained from the CEC-2017 benchmark suite^12–14^. Friedman’s statistics have been carried out considering the significance level $\:\alpha\:$ to be 0.05. By applying Friedman’s test, it ranks the employed HA. The numerical rank in the extent [1, N] has been assigned to all the present-day approaches, based on the result reported from different benchmark suites, as represented in Table 9. The null hypothesis is rejected only if Friedman’s score exceeds the critical value observed from the f-table. The outcome is computed to be 35.915, implying a greater value than the observed critical value (4.365), signifying refusal of the null hypothesis, indicative of the existence of some variation among the optimization methods^36^. To check whether the suggested LS-SSA outperforms other present-day approaches, Holm’s test is conducted based on the experiential $\:\text{P}\text{R}\text{V}\text{L}$ value^37^. From Table 10, it can be inferred that the observed $\:\text{P}\text{R}\text{V}\text{L}$ is less than the $\:\frac{\alpha\:}{\left(\mathcal{T}\mathcal{A}-\mathcal{i}\right)}$ values, which tends to exclude the hypothesis. Finally, the Wilcoxon signed-rank test is being performed pair-wise between the suggested LS-SSA and present-day methods as presented in Table 11. It is clear from the observed outcomes that LS-SSA performs significantly in both dimensions 10 and 30 when compared to other algorithms. Henceforth, the proposed LS-SSA as a controlling algorithm endorses its superiority with better performance and efficacy.

**Table 9:** Mean value ranking of present-day approaches.

	ALO	MFO	SCA	WOA	SSA	LS-SSA
f1	1.762E + 04(6)	1.527E + 04(5)	1.450E + 04(4)	3.485E + 03(3)	2.452E + 03(2)	**2.689E + 02(1)**
f2	1.714E + 13(6)	7.095E + 11(5)	2.913E + 11(4)	9.486E + 09(3)	4.160E + 08(2)	**3.444E + 08(1)**
f3	2.010E + 04(6)	1.882E + 04(5)	1.734E + 04(3)	1.940E + 04(4)	4.003E + 03(2)	**1.344E + 03(1)**
f4	7.376E + 01(6)	6.775E + 01(4)	6.871E + 01(5)	4.746E + 01(3)	3.064E + 01(2)	**1.538E + 01(1)**
f5	4.508E + 09(5)	3.998E + 09(4)	4.608E + 09(6)	2.684E + 08(3)	2.222E + 08(2)	**2.102E + 06(1)**
f6	1.570E + 04(5)	1.628E + 04(6)	1.185E + 04(4)	3.187E + 03(2)	4.591E + 03(3)	**2.940E + 02(1)**
f7	1.980E + 08(6)	1.848E + 08(5)	1.555E + 08(4)	3.572E + 07(3)	8.783E + 06(2)	**1.403E + 05(1)**
f8	-2.717E + 02(5)	-2.115E + 02(6)	-4.009E + 02(3)	**-5.607E + 02(1)**	-2.740E + 02(4)	-4.470E + 02(2)
f9	1.461E + 04(5)	1.462E + 04(6)	1.398E + 04(4)	3.033E + 03(3)	2.883E + 03(2)	**4.884E + 02(1)**
f10	2.022E + 01(4)	2.088E + 01(6)	2.005E + 01(3)	**6.377E + 00(1)**	2.060E + 01(5)	1.973E + 01(2)
f11	5.112E + 00(6)	4.881E + 00(4)	4.691E + 00(5)	1.593E + 00(2)	1.758E + 00(3)	**1.127E + 00(1)**
f12	3.278E + 09(5)	2.001E + 09(4)	3.573E + 09(6)	6.234E + 07(3)	5.123E + 07(2)	**3.537E + 04(1)**
f13	4.158E + 09(5)	4.070E + 09(6)	2.887E + 09(4)	2.426E + 08(3)	1.045E + 08(2)	**1.178E + 05(1)**
f14	7.518E-03(2)	1.309E-01(6)	**6.721E-03(1)**	1.000E-02(3)	1.593E-02(4)	7.152E-02(5)
f15	1.573E + 00(5)	3.087E + 01(6)	4.195E-01(2)	6.969E-01(3)	9.753E-01(4)	**1.936E-01(1)**
f16	6.321E + 02(5)	6.980E + 02(6)	3.826E + 02(3)	5.115E + 02(4)	9.101E + 01(2)	**6.227E + 00(1)**
f17	2.091E + 04(5)	1.988E + 04(4)	1.438E + 04(3)	4.271E + 08(6)	5.608E + 03(2)	**3.215E + 02(1)**
f18	1.805E + 02(6)	1.298E + 02(4)	1.322E + 02(5)	7.223E + 01(3)	6.093E + 01(2)	**5.023E + 01(1)**
f19	1.720E + 02(6)	1.475E + 02(5)	1.465E + 02(4)	6.999E + 01(3)	6.509E + 01(2)	**4.830E + 01(1)**
f20	8.942E + 05(4)	9.093E + 05(5)	7.218E + 05(3)	6.012E + 04(2)	9.626E + 04(6)	**2.556E + 03(1)**
f21	5.502E + 09(5)	3.499E + 09(4)	6.836E + 09(6)	5.965E + 08(3)	2.139E + 08(2)	**1.235E + 06(1)**
f22	1.544E + 19(5)	1.779E + 19(6)	1.064E + 19(4)	3.318E + 17(3)	7.226E + 16(2)	**2.487E + 13(1)**
f23	-5.280E + 02(2)	-3.933E + 02(6)	-5.253E + 02(3)	**-8.298E + 02(1)**	-4.370E + 02(5)	-4.709E + 02(4)
f24	1.262E + 04(6)	1.155E + 04(5)	8.200E + 03(4)	2.081E + 03(3)	1.777E + 03(2)	**8.837E + 02(1)**
f25	4.061E + 02(6)	3.453E + 02(4)	3.590E + 02(5)	1.984E + 02(3)	1.860E + 02(2)	**1.127E + 02(1)**
f26	-1.065E + 04(3)	-9.163E + 02(6)	-2.693E + 04(2)	**-3.462E + 04(1)**	-3.485E + 03(5)	-3.754E + 03(4)
f27	7.153E + 04(6)	7.092E + 04(5)	4.677E + 04(4)	1.025E + 04(2)	1.176E + 04(3)	**1.692E + 03(1)**
f28	-3.727E + 00(5)	-4.052E + 00(3)	-3.774E + 00(4)	**-8.727E + 00(1)**	-3.459E + 00(6)	-4.638E + 00(2)
	**5.04**	**5.04**	**3.86**	**2.68**	**2.93**	**1.46**

**Table 10:** Outcome from Holms Test.

	Z-value	$\:\text{P}\text{R}\text{V}\text{L}$	$\:\frac{\alpha\:}{\left(\mathcal{T}\mathcal{A}-\mathcal{i}\right)}$	Hypothesis
ALO	-7.46	0.00004	0.01	Omitted
MFO	-6.82	0.00004	0.0125	Omitted
SCA	-5	0.00004	0.016667	Omitted
WOA	-2.29	0.01101	0.025	Omitted
SSA	-2.86	0.00212	0.05	Omitted

**Table 11:** Non-parametric outcomes from the Wilcoxon Signed-Rank test.

Dimension	Comparison	S^+^ Values	S^−^ Values	$\:{\uprho\:}$ value	Null Hypothesis	Sign	Difference
10	LS-SSA vs. SSA	405	1	1.49E-08	Rejected	+	Large
	LS-SSA vs. MFO	406	0	7.45E-09	Rejected	+	Large
	LS-SSA vs. ALO	386	20	2.76E-06	Rejected	+	Large
	LS-SSA vs. SCA	378	28	1.11E-05	Rejected	+	Large
	LS-SSA vs. ACO	282	124	7.35E-02	Rejected	+	Medium
	LS-SSA vs. WOA	356	50	2.18E-04	Rejected	+	Large
30	LS-SSA vs. SSA	375	3	7.45E-08	Rejected	+	Large
	LS-SSA vs. MFO	405	1	4.46E-06	Rejected	+	Large
	LS-SSA vs. ALO	376	30	1.51E-05	Rejected	+	Large
	LS-SSA vs. SCA	371	35	3.18E-05	Rejected	+	Large
	LS-SSA vs. ACO	219	187	7.28E-01	Rejected	+	Small
	LS-SSA vs. WOA	317	89	8.23E-03	Rejected	+	Medium

## Brain MRI classification over proposed LS-SSA with deep CNN

The following subsection outlines the description of the dataset and includes the application of LS-SSA in optimizing the parameters of CNN.

### Dataset outline

The dataset utilized to evaluate the proposed LS-SSA as an application to tune the hyperparameters of CNN has been employed from KAGGLE^15^. It consists of Brain MRI scans for multi-class classifying tumors. Table 12 summarizes the dataset description.

**Table 12:** Dataset Description^15^.

Class Label	Tumor	Training Set	Testing Set
0	Glioma	1321	300
1	Meningioma	1339	306
2	No	1595	405
3	Pituitary	1457	300

The dataset consists of Brain MRI, which primarily includes the following classes:

#### Glioma tumor

This class includes the scans of patients classified with glioma disorder that come from the glial cells extant in the brain and spinal cord. The dataset consists of 1321 images for training set and 300 testing set images of Glioma Tumor.

#### Meningioma tumor

This sort of tumor is usually a benign, slow-growing tumor that arises from the protective sheath meninges, which shield the brain and spinal cord. The dataset consists of 1339 training set scans and 306 scans for the testing set of Meningioma Tumor.

#### No tumor

The class comprises the test of the patients identified with no tumor. The dataset consists of 1595 images for the training set and 405 testing set of No Tumor.

#### Pituitary tumor

**Fig. 6:**
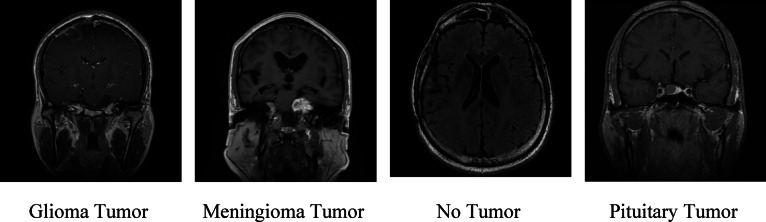
Brain MRI scans classifying the tumor^15^.

This sort of tumor is typically benign and develops in a small gland known as the pituitary gland, located at the base of the brain. The dataset consists of 1457 training set scans and 300 scans for testing sets of Pituitary Tumors. Figure 6 represents the various tumor classification scans of brain MRI.

### CNN and its structure

**Fig. 7:**
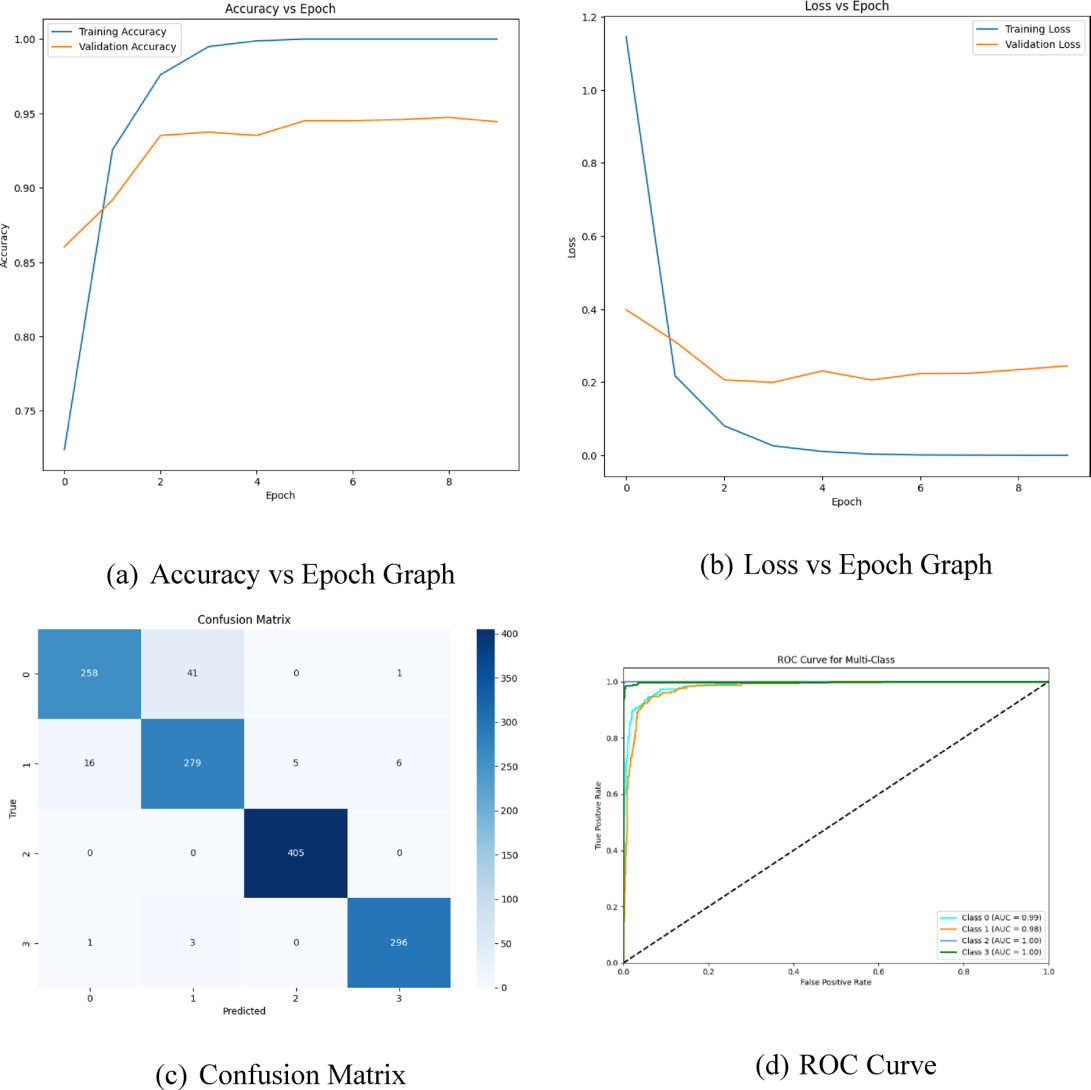
Graphical representation of findings obtained from the suggested LS-SSA-CNN.

CNN is primarily a class of deep neural networks uniquely cast off in the domain of medicine^38,39^. It is well-defined as the cornerstone for vision applications as its structure is defined in such a way that it can adapt and learn the spatial hierarchical features of the input images, making it more well-suited for the motive of object detection, image classification, image segmentation, facial recognition, medical image analyzing, video analyzing, and natural language processing^40,41^. Regardless of their notable results in the field of medical imaging, the performance of CNNs is highly synchronized to the initialization of initial parameters and optimizing their hyperparameters^42,43^. For training the CNN, the hyperparameters serve the crucial role in detecting the model’s interpretability to handle and generalize the new unseen data, failing to which may result in artefacts and overfitting^44^. The hyperparameters are the structural units that define the modular configuration of CNN^45^. It includes the count of convolutional layers, count of dense neurons, count of filters, filter size, batch size, and max pooling layers, which are the structural units of CNN and define the CNN model. This study aims to provide a systematic approach to hyper-tune the parameters for CNN, focusing mainly on two parameters, namely the count of filters and dense neurons^46,47^. These parameters are explored within a defined search radius to exploit the solution to achieve the optimum that enhances the efficacy of the manifest model. In this study, we have considered a SI-based metaheuristic approach, namely SSA, but the major concern associated with the method is prone to confined optima stagnancy. To prevent such issues and to converge timelessly to the global optimum, hybrid LS-SSA is utilized. The parameters, namely dense neurons count and filter count, are randomly positioned in the search bound in [16,64] and [64,256]. The parameters are assessed by utilizing the fitness function and are modified over the optimization process concerning the foremost salp. The fitness functions aim to diminish the error rate over ten epochs, tuned by the suggested LS-SSA. The graphical representation of findings obtained from the suggested LS-SSA-CNN is depicted in Fig. 7. The experimental outcomes concerning to hybrid SSA and proposed LS-SSA integrated with CNN along with modern-day approaches, are presented in Table 13.

**Table 13:** Experimental Results.

	CNN	MFO-CNN	WOA-CNN	SCA-CNN	ACO-CNN	SSA-CNN	LS-SSA-CNN
MIN RMSE	0.0844	0.0533	0.0615	0.0901	0.0609	0.0563	**0.0559**
AVG	0.1871	0.1367	0.1381	0.1511	0.1434	0.1397	**0.1371**
STD	0.0681	0.0644	0.0649	0.061	0.0709	0.0683	**0.068**
ACCURACY	93.35	94.43	94.28	93.21	93.82	94.1	**94.43**
ERROR RATE	6.65	5.57	5.72	6.79	6.18	5.9	**5.57**
PRECISION	94.41	92.96	91.65	96.07	95.53	94.47	**97.02**
SPECIFICITY	94.33	98.17	98.22	97.76	97.98	98.1	**98.17**
SENSITIVITY	91.05	93.96	94.18	92.69	93.35	93.72	**93.96**
F1-SCORE	92.69	93.45	92.89	94.34	94.42	94.09	**95.46**

## Conclusion

This article suggests an enhanced sort of SSA that incorporates the localized search agents as LS-SSA. The main aim of the study is to exploit the explorative potency of the standard SSA through a new evolutionary operator named LS. At times, SSA fails to obtain the optimum solution as it is stagnant in the confined minima with penurious convergence rate and condensed sort of solutions. By incorporating the LS approach to standard SSA, a broad range of finer candidate solutions can be achieved by refining the initial solutions. As a result, the uniqueness of the suggested LS-SSA is established through a series of statistical analyses carried out by comparing five present-day methods. The sturdiness and proficiency of the suggested LS-SSA model are assessed by using the twenty-eight constraints benchmark suite accessible in IEE-CEC-2017. Analyzing the stated result regarding function types such as unimodal, multimodal, hybrid and composite, the suggested LS-SSA dominates its competence over SSA and present-day techniques. Finally, to address the problems of CNN, like data overfitting, sensitivity to artefacts and inadequate selection of initial parameters, the suggested LS-SSA as an application has been used to optimize the hyperparameters of CNN on the standard brain MRI scan dataset. The effectiveness of the present-day models is determined in terms of greater accuracy of 94.43%, lower error rate of 5.57%, min RMSE of 0.0559, avg of 0.1371, SD of 0.068, greater value for precision of 97.02%, specificity of 98.17%, sensitivity of 93.96% and f1-score of 95.46%. From the reported outcome, enhanced accuracy, lower SD, lower RMSE, and improved average signify the exploration power of the approach with the optimized hyperparameters. As the proposed LS-SSA model falls into the set of stochastic methods, the results may vary across the different runs of the algorithms, and the major setback for any metaheuristic algorithm is that there is no common mathematical framework for assessing the efficacy.

In future work, the suggested LS-SSA is planned to utilize diversified domains of engineering like autonomous vehicles, natural language preprocessing, robotics, fraud detection, and stock market prediction. Further, the same approach is suitable for ML-IoT-based healthcare management.

## Data Availability

The datasets used and analyzed during the current study are available from the corresponding author upon reasonable request.
